# Lid Margin Microbiome in Stevens-Johnson Syndrome Patients With Lid Margin Keratinization and Severe Dry Eye Disease

**DOI:** 10.1167/iovs.65.6.28

**Published:** 2024-06-18

**Authors:** Swati Singh, Moumi Maity, Swapna Shanbhag, Kotakonda Arunasri, Sayan Basu

**Affiliations:** 1 Center for Ocular Regeneration (CORE), L. V. Prasad Eye Institute, Hyderabad, Telangana, India; 2 Brien Holden Center for Eye Research (BHERC), L. V. Prasad Eye Institute, Hyderabad, Telangana, India; 3 Shantilal Shanghvi Cornea Institute, L. V. Prasad Eye Institute, Hyderabad, Telangana, India

**Keywords:** stevens-johnson syndrome (SJS), microbiome, *corynebacteria*, lid margin keratinization (LMK)

## Abstract

**Purpose:**

The current study evaluated the lid margin microbiome of keratinized lid margins of patients with chronic Stevens-Johnson syndrome (SJS) and compared it with healthy controls and historically reported lid margin microbiome of patients with meibomian gland dysfunction (MGD).

**Methods:**

Eyelid margin swabs of 20 asymptomatic adults (mean age = 29 ± 12 years) and 10 patients with chronic SJS (mean age = 31.2 ± 14 years) with lid margin keratinization were sequenced using next generation of 16S rDNA V3 to V4 variable region. Within SJS, the keratinized lid margin microbiome was compared with adjacent eyelid skin.

**Results:**

All patients had obstructive MGD, and mean Schirmer I value was 2.8 ± 1.9 mm. The phyla were similar in two groups, whereas at the genera level, an increase in the relative abundance of *Corynebacterium*, *Haemophilus*, *Azotobacter*, and *Afipia* and a decrease of *Acinetobacter* was noted in SJS compared to healthy lid margins. SJS-associated microbiota displayed lesser diversity and more heterogeneity than healthy controls. The Principal Components Analysis (PCA) plot revealed wide separation in the SJS and the control groups. Correlational network analysis revealed *Corynebacterium* and *Sphingomonas* forming a major hub of negative interactions with other bacterial genera in the SJS group. Significant differences exist in the prevalent genera between keratinized lid margins and historically reported meibum microbiome of patients with MGD. In addition, the eyelid skin of patients with SJS had predominant *Staphylococcus*, whereas *Corynebacterium* and *Pseudomonas* were more in the keratinized lid margins compared to the eyelid skin microbiome.

**Conclusions:**

Lid margin microbiome is significantly altered in the keratinized lid margins of patients with SJS compared to the eyelid skin of patients with SJS, normal lid margins, and patients with MGD.

Lid margin keratinization (LMK), a sequela of Stevens-Johnson syndrome (SJS), causes ocular surface inflammation and corneal epitheliopathy due to the lid wiper effect.^1^ In eyes with LMK, the transitional zone between the eyelid skin and the conjunctival epithelium is replaced by keratinized stratified squamous epithelium. However, its pathophysiology is poorly understood. One of the proposed theories suggests that the source of LMK could be the hyperkeratinized epithelium arising from the meibomian gland ductal orifices and altered lid microbiota.^1^ Clinically, the keratinization involves the meibomian gland’s orifices, and these patients have a severe obstructive variety of meibomian gland dysfunction (MGD) with no expressible meibum.^1^ Lid margin bacterial microbiota has been implicated as one of the important causative factors for MGD in general,^2^^–^^4^ but its role in LMK has not been studied yet.

The changes in the ocular surface microbiome of the conjunctival microbiota have been reported in dry eye disease, contact lens wear, chronic ocular graft-versus-host disease, and also in SJS.^5^^–^^12^ The ocular surface microbiome in patients with SJS is known to be altered with a predominance of pathogenic bacteria. The ocular surface microbiome from the inferior conjunctival fornix of patients with SJS has a higher proportion of pathogenic microorganisms, including *Pseudomonas spp.*, *Staphylococcus spp.*, *Streptococcus spp.*, and *Acinetobacter spp*., assessed using conventional culture techniques.^8^ It is important to note that the ocular surface and lid margin microbiome show significant differences in species diversity and are not interchangeable. The lid margin microbiome studied from tissue obtained from eyelid surgery has shown more of the *Corynebacterium* and *Pseudomonas* species and less of the *Staphylococcus* and *Streptococcus* species.^11^ On the ocular surface, the meibum and conjunctival microbiome show similarities in young, healthy adults but differences in elderly subjects.^12^ However, these observations have not been looked at in patients with SJS.

The role of lid microbiome in LMK remains largely unexplored, with few studies on healthy or keratinized lid margins using high throughput next-generation sequencing (NGS) methods that can generate large amounts of data. The current study, therefore, evaluated the lid margin microbiome in patients with LMK and compared it with healthy controls as well as historically reported lid margin/meibum microbiome of patients with MGD using NGS.

## Methods

This prospective study included 20 healthy asymptomatic young adults and 14 consecutive patients with SJS with LMK who were due to undergo mucous membrane grafting (MMG) surgery. All clinical procedures were done by one of the authors (S.S). The Institutional Ethics Committee approved the study and adhered to the tenets of the Declarations of Helsinki. The severity of ocular surface changes was graded according to the Sotozono's grade scoring system.^13^ Patients who had undergone MMG or any other eyelid intervention in the past, as well as those with active ocular infection or hordeolum, were excluded. None of the patients had a history of recent use of topical or systemic antibiotics in the past 4 weeks. Healthy subjects were matched to the SJS group for age and gender and recruited from among the hospital staff volunteers who had no ocular complaints or ocular signs of allergy, inflammation, or any other ocular surface diseases. None of the subjects were contact lens users.

### Sample Collection

**Figure 1.**
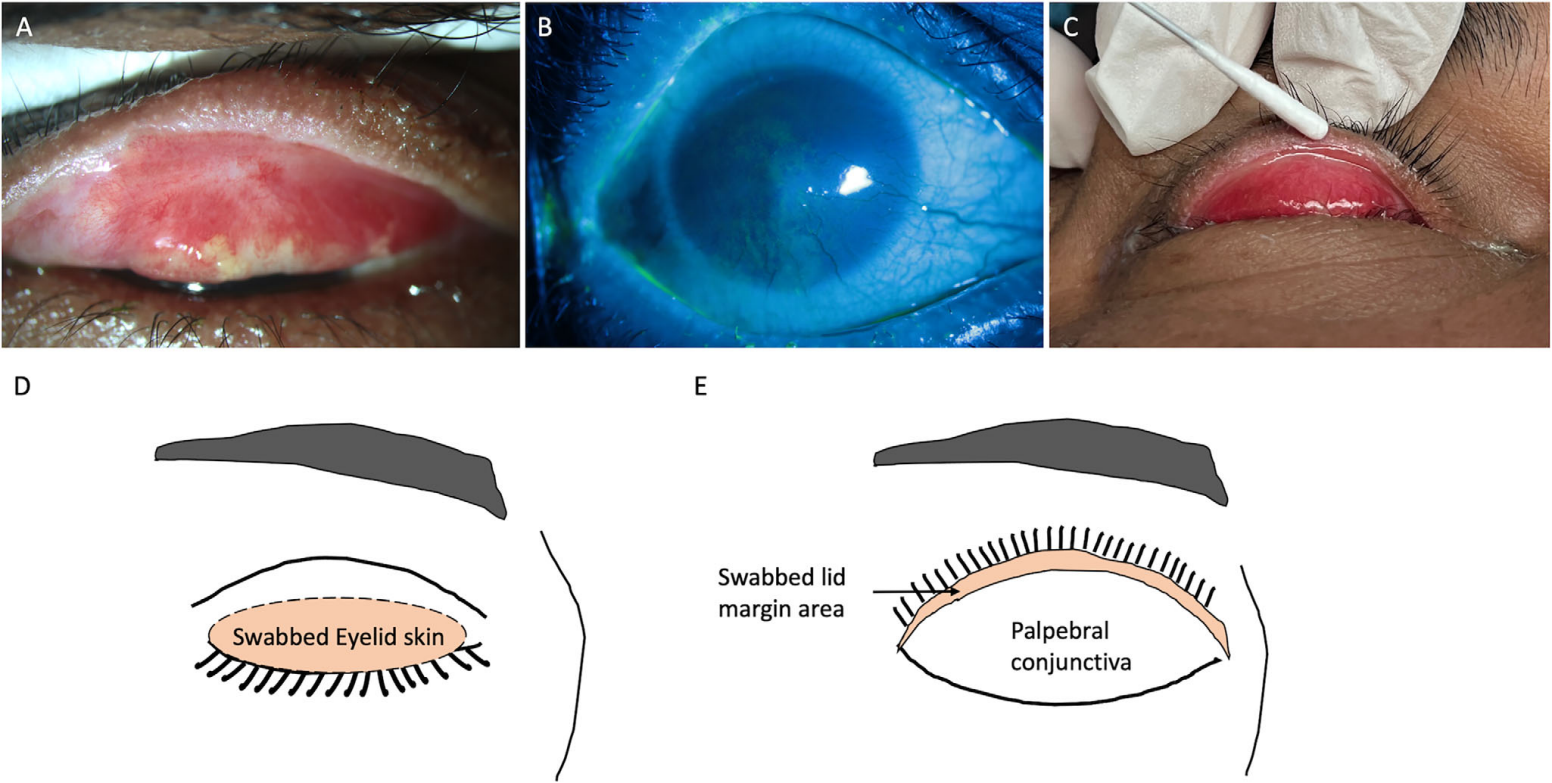
(**A**) The everted left upper eyelid shows lid margin keratinization with tarsal conjunctiva scarring and congestion. (**B**) The cornea shows diffuse punctate keratitis with superficial vascularization and limbal stem cell deficiency. (**C**) Technique of collecting a sample from a keratinized segment of lid margin using a sterile cotton swab. (**D,** **E**) Schematics showing the site of sample collection eyelid skin away from the lashes (**D**, *highlighted area* of closed upper eyelid) and keratinized lid margin (**E**, *highlighted* lid margin area of the everted upper eyelid).

After obtaining informed consent, the individuals (healthy subjects and patients with SJS) were asked to lie on the operating table under an operating microscope. A drop of proparacaine (0.5%) was instilled into the conjunctival sac for better patient cooperation and reduced blinking while touching the lid margin to avoid contamination with tears. The upper eyelid was gently everted with sterile gloved hands. The keratinized lid margin area (the shaded area in Fig. 1E) was swabbed with sterile flocked dry swabs (HiMedia Laboratories, Mumbai, India). The keratinized lid margin area denotes the lid wiper region that comes in contact with the globe and extends from the meibomian gland openings to the subtarsal fold. Swabs were rubbed with pressure across the keratinized lid margin (see Fig. 1) from the medial to the lateral end and then from the lateral to the medial end. The samples were collected in the operation theater that are maintained sterile with positive pressure air outflow. Care was taken to avoid any contact with the adjacent eyelid skin or bulbar conjunctiva. Separate swabs collected eyelid skin microbiome from both upper and lower eyelids (see Fig. 1). The sterile dry swabs (HiMedia Laboratories, Mumbai, India) were rubbed over the eyelid skin 20 to 30 times in the pre-tarsal area and the skin near the lid margin. The swabs were immediately placed in sterile PBS-filled tubes and transported to the laboratory in an ice box to be stored at −20 degrees until further processing. All samples were collected by one investigator (author S.S). Samples were collected randomly from the patients with SJS and the control group for over 6 months. The same batch of swabs and DNA extraction kits were used.

### 16S rDNA Sequencing and Microbiome Generation

DNA extraction was performed using the “QIAmp DNA Mini Kit” from Qiagen (Cat No: 51304; Supplementary File S1). Concentration of DNA samples were measured with Nanodrop ND-100 Spectrophotometer (NanoDrop Technologies, Wilmington, DE, USA) and Qubit 4 Fluorometer (Thermo Fisher Scientific, Indianapolis, IN, USA), whereas DNA quality was assessed with Agarose Gel Electrophoresis using 1% Agarose Gel and observed on Syngene Gel Documentation System G: Box. Illumina high-throughput sequencing (MiSeq Illumina Sequencing Platform) was used to sequence the 16S rDNA V3 to V4 hypervariable region of all bacteria in lid margin swab samples. Paired-end raw sequence reads (FASTQ; Supplementary File S2) were assessed for base quality and contamination by sequencing artifacts. Quality profiling and error estimation of sequence reads was performed with R package DADA2. Trimming of adapters and poor-quality sequences was performed for paired sequence reads with Trim Galore. DADA2 workflow was used to filter, merge, denoise, and filter chimeric amplicons to obtain Amplicon Sequence Variant (ASV). DADA2 workflow was used to perform the taxonomic classification of ASV by using Silva version 138 reference annotation. Kraken2 and Bracken are used to align the filtered reads to reference 16s rRNA annotation from Silva version 138. An interactive hierarchical chart representing the taxonomic classification was generated with the Krona tool. Abundance plots for taxonomic classification were generated with R packages phyloseq and ampvis2. For excluding the environmental and lab-related contaminants as the source of the bacteria, we ran “blank” controls (i.e. a PCR performed with the same Illumina-suggested primers but no DNA template). The obtained data were subtracted using microsecond (a read-subtraction tool) from the test and healthy control samples.^14^

#### Differential Abundance and Statistical Analyses

Differential network analysis was performed for comparisons between the two groups. The Principal Components Analysis (PCA) and bar-plot figures were generated using R (version R-3.6). The heatmap figures were generated using pheatmap and ComplexHeatmap packages. Bacteria-bacteria interactions among the bacterial genera of the healthy control group and the SJS group were assessed by CoNet network analysis.^15^

## Results

### Normal Lid Margin

**Figure 2.**
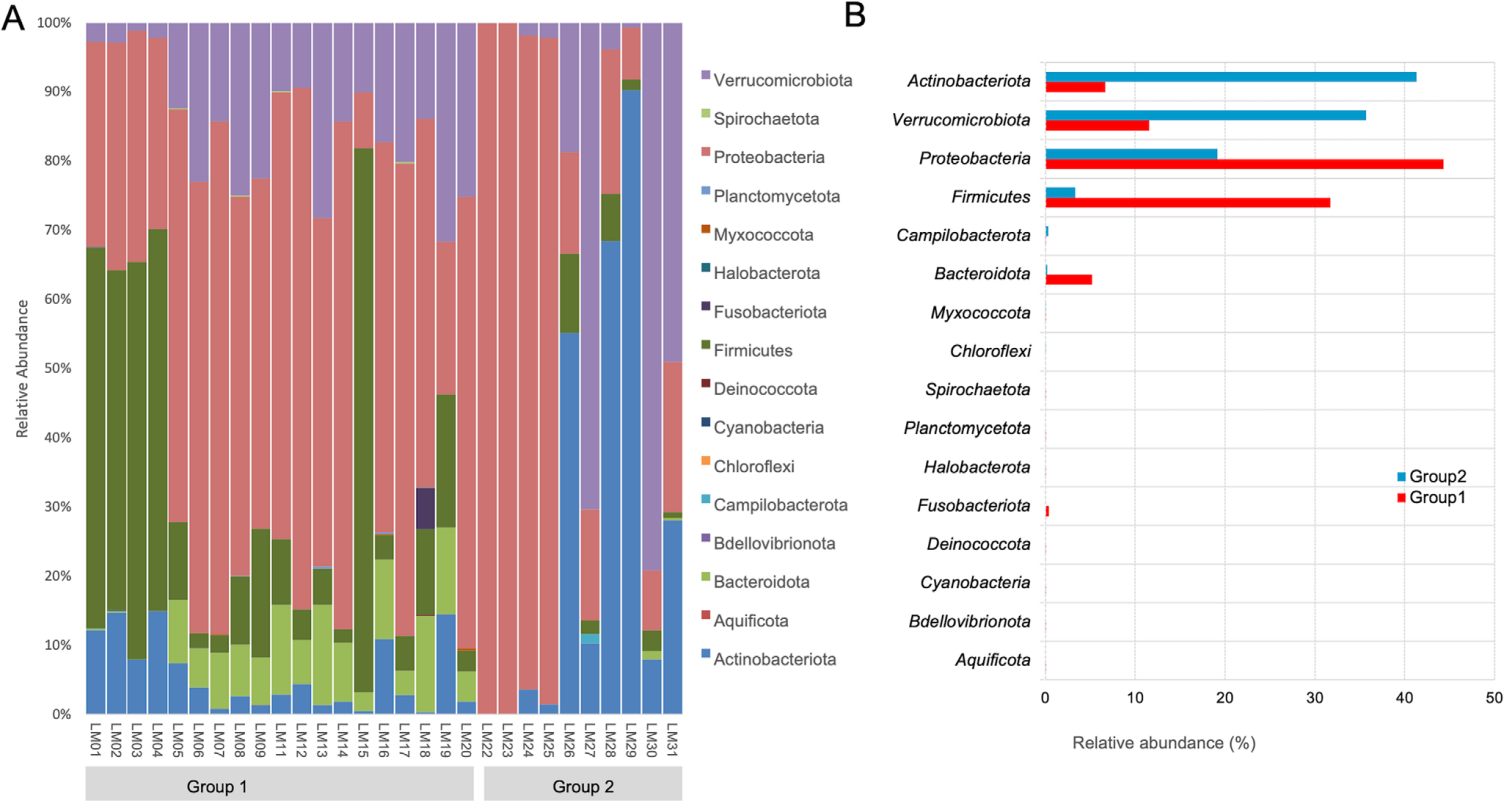
(**A,** **B**) Relative abundance and box plot of phyla distribution in all the samples of both groups (group 1 = healthy lid margins and group 2 = SJS lid margins).

**Figure 3.**
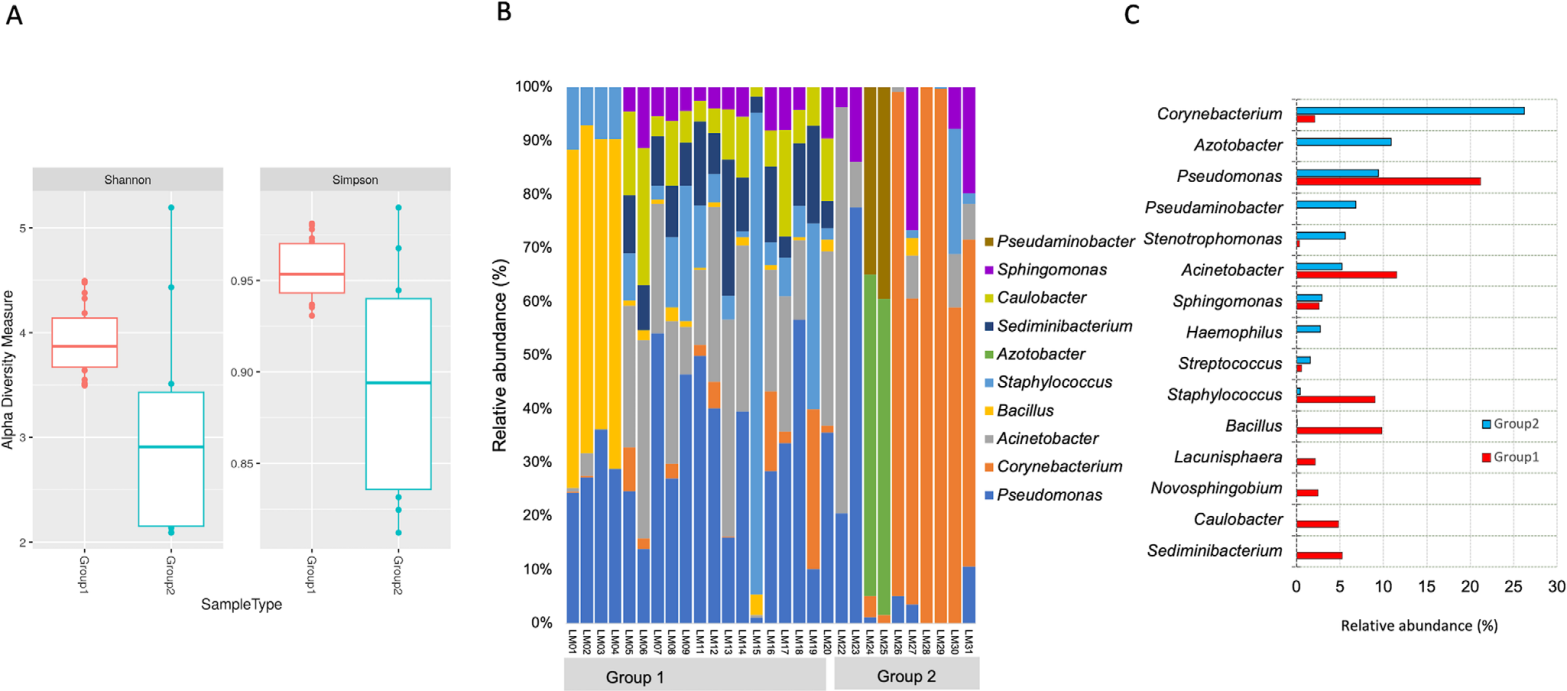
(**A**) Shannon and Simpson diversity indices show more diverse flora in SJS lid margins than healthy lid margins. (**B,** **C**) Relative abundance of genera in both groups’ samples (group 1 = healthy lid margins and group 2 = SJS lid margins).

The mean age of 20 healthy individuals (13 men) was 29 ± 12 years and matched the SJS group. None of them had any ocular complaints and had normal slit lamp ocular surface evaluation (no signs of blepharitis). The five major phyla identified across the normal lid margins include Proteobacteria, Bacteroidota, Actinobacteriota, Firmicutes, and Verrucomicrobiota (Fig. 2), constituting more than 95% of lid margin microbiota. The prevalent genera include *Pseudomonas*, *Acinetobacter*, *Staphylococcus*, *Sediminibacterium*, *Bacillus*, and *Caulobacter* (Fig. 3).

### SJS Lid Margin

#### Clinical Details

Ten patients with chronic SJS with a mean age of 31.2 ± 14 years (range = 18–48 years, 7 men) were included. Of 14 enrolled patients with SJS, 4 lid margin swabs did not show enough nucleic acid material of good quality and, hence, were not processed further. The etiology of SJS (*n* = 10) was drug-induced in four, post-viral fever in two, and unknown in the other four patients. The mean SJS duration from the acute attack was 14.2 ± 10.3 years (6–27 years). All patients had eyelid margin keratinization with a mean Sotozono’s scoring system of 9.7 ± 1.8, 1.2 ± 1.1, and 8 ± 5.6 for eyelid, conjunctival, and corneal complications, respectively. All patients had obstructive meibomian gland disease with posterior migration of the mucocutaneous junction. The mean Schirmer I value was 2.8 ± 1.9 mm at 5 minutes. None of the patients were using any contact lenses. Three patients had undergone ocular surgeries - electroepilation (*n* = 2), and amniotic membrane graft (*n* = 1) 1 year before the lid margin MMG. All of them were on lubricants, and 3 used low-potency steroid drops for 3 weeks. These 3 patients were asked to discontinue drops 1 week prior to the procedure. The lid margin showed keratinization in all margins extending onto the lid wiper conjunctiva. Meibomian glands were obstructed in all with no expressibility.

#### Lid Margin Microbiome

##### Alpha and Beta Diversity

**Figure 4.**
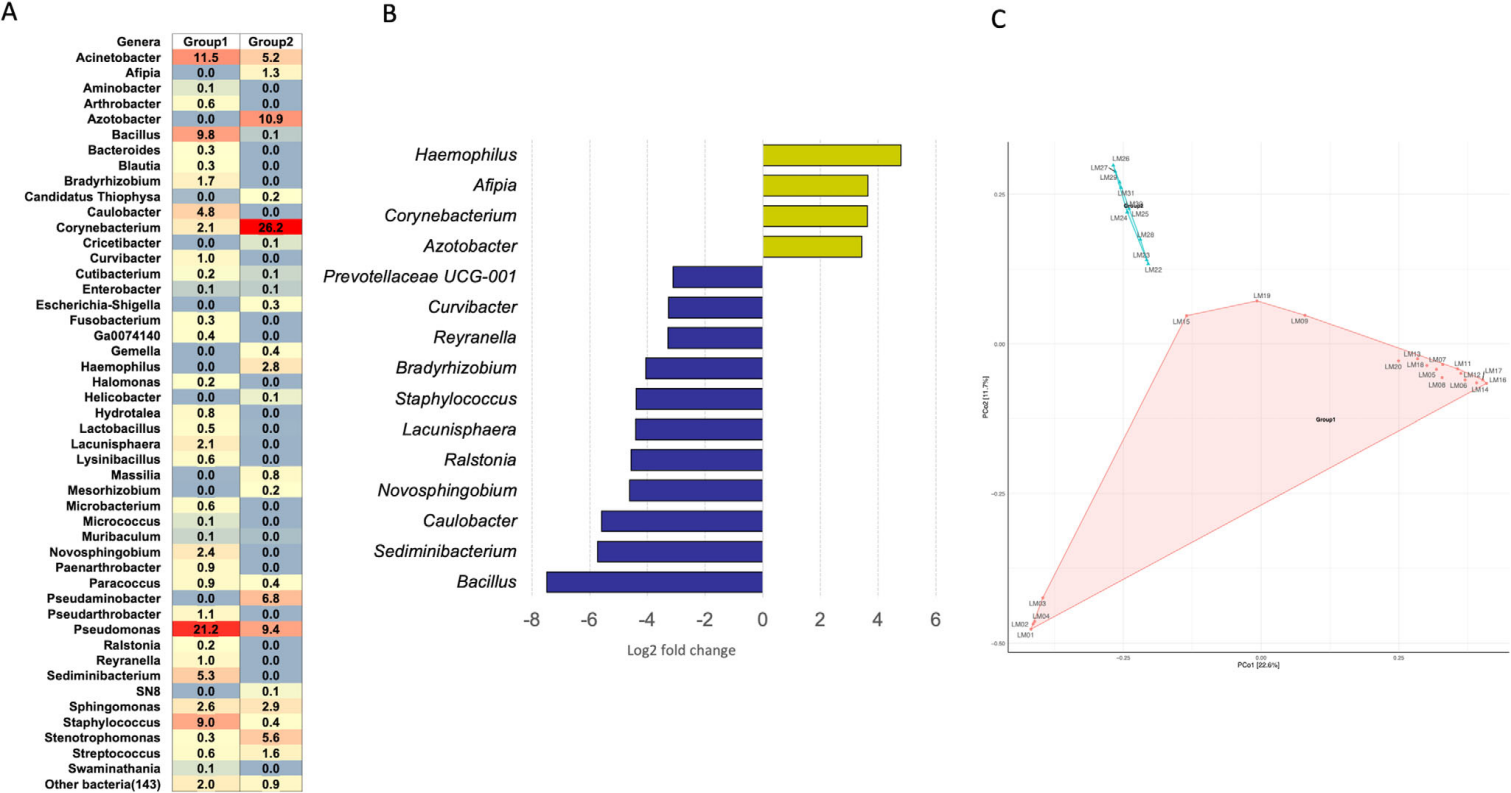
(**A**) Heatmap showing percentage relative reads abundance between the two groups. The percentage relative abundance is indicated by the gradient of *red* to *blue*. (**B**) Significantly differential abundance of the top 15 bacterial genera (Log 2 fold change) in SJS versus healthy lid margins. (**C**) PCoA plot showing dissimilarity between the two group clusters separated by a huge gap.

Shannon and Simpson indices revealed more case-to-case variability in SJS than healthy lid margins (see Fig. 3). Beta diversity as measured by Principal coordinates (PCoA) analysis revealed significant dissimilarity between SJS and control samples as there was a huge separation of two clusters on PCoA plots (Fig. 4C).

The 5 major phyla identified across the SJS lid margins include Proteobacteria, Verrucomicrobiota, Actinobacteriota, Firmicutes, and Bacteroidota (see Fig. 2), which constituted more than 95% of lid margin microbiota. The prevalent genera in both groups include *Corynebacterium*, *Sphingomonas*, *Pseudomonas*, *Acinetobacter*, and *Streptococcus* (see Fig. 3). The heat maps at the phylum (see Fig. 2A) level show the relative abundance of the respective organisms for each individually tested lid margin sample of control subjects and patients with SJS. Thirty-six taxa were significantly different in SJS lid margins compared to the control group (Supplementary File S3). The abundance of *Corynebacterium*, *Azotobacter*, *Haemophilus*, and *Afipia* was significantly higher in SJS lid margins compared to controls, as shown in the Log_2_ fold change comparison of the two groups (Fig. 4B). *Pseudomonas*, *Acinetobacter*, *Sediminibacterium*, *Bacillus*, and *Staphylococcus* significantly decreased in keratinized lid margins (see Fig. 4B). A comparison of the prevalent bacterial genera between keratinized lid margins and meibum microbiome reported in the literature was tabulated (Table).

**Table.** 

	Healthy Lid Margins	Keratinized Lid Margins	Meibum From MGD^*^
Prevalent genera	*Pseudomonas, Acinetobacter, Staphylococcus, Sediminibacterium Caulobacter*	*Corynebacterium, Sphingomonas, Pseudomonas, Acinetobacter, Streptococcus*	Pseudomonas, Cutibacterium, Campylobacter, Corynebacterium, Rubrobacter

* Predominant genera as per the data from Reference no. 4, Zhao et al. 2020.

##### Network Analysis

**Figure 5.**
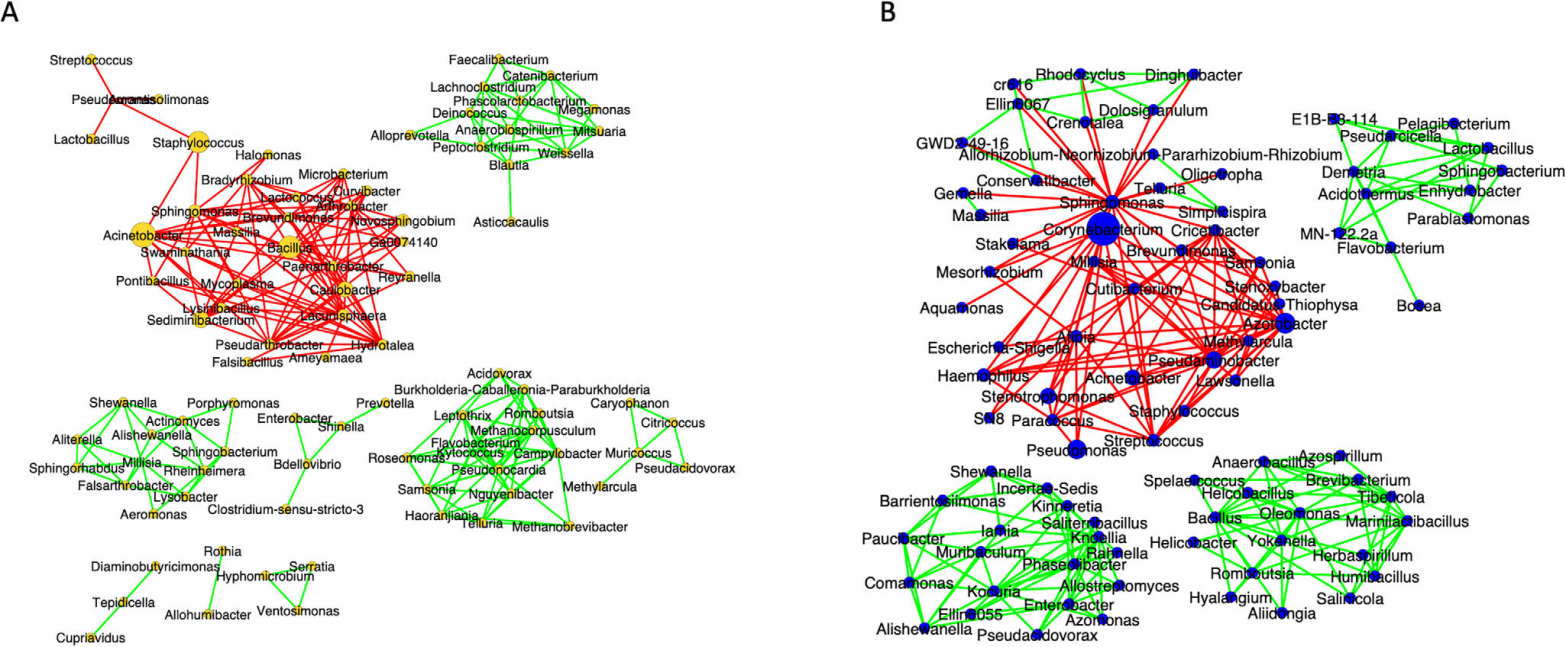
Correlational network analysis of group 1 (**A**) versus group 2 (**B**). The *green edges* indicate the positive interactions of these bacteria, and the negative interactions are indicated by the *red edges* (group 1 = healthy lid margins and group 2 = SJS lid margins).

Correlational network analysis depicted the positive and negative interactions among the bacterial genera.^13^ In both groups, the predominant bacterial genera formed negative interactions with other bacterial genera, indicating competition for nutrition and other resources (Fig. 5). In the healthy group, the negatively interacting bacteria and positively interacting genera formed separate clusters without any interception. Conversely, in the SJS group, the genera *Corynebacterium* and *Sphingomonas* formed a major hub of negative interactions with other bacterial genera that were interacting positively with each other.

#### Eyelid Skin Microbiome

**Figure 6.**
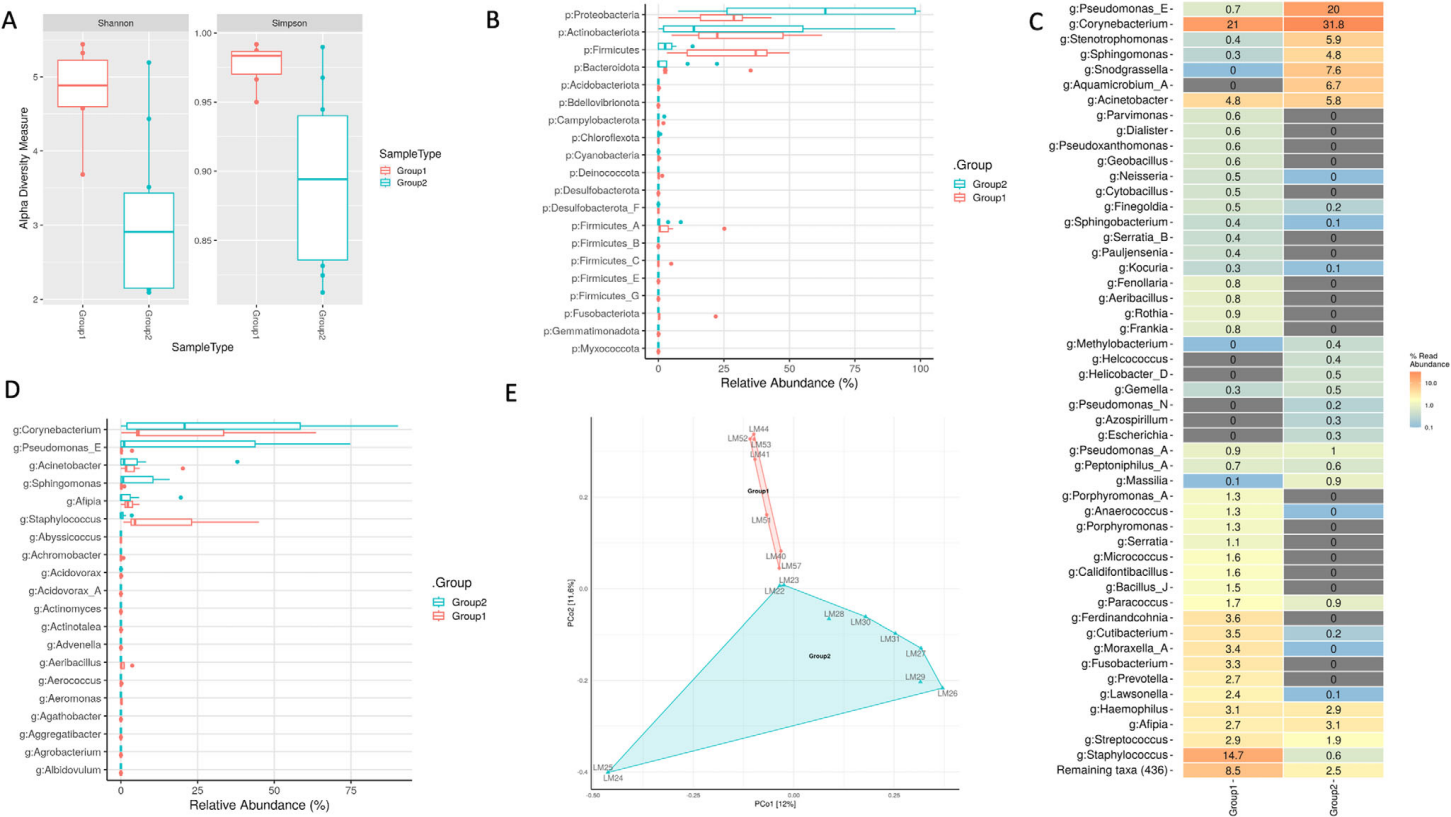
Comparison of eyelid skin (labeled as group 1) with lid margin microbiome (labeled as group 2) of patients with SJS. (**A**) Shannon and Simpson diversity indices show more diverse flora in the lid margins than in the eyelid skin. (**B**, **D**) Relative abundance of phyla and genera in both groups’ samples. (**C**) Heatmap showing percentage relative reads abundance between the two groups. (**E**) PCo_2_ plot showing distinct clusters of the two groups separated by a small gap.

Of 14 enrolled patients with SJS, only 7 lid margin swabs showed enough nucleic acid material of good quality; hence, the remaining 7 were not processed further. Eyelid skin of patients with SJS showed differences in microbiota from the lid margin microbiome. Firmicutes were the predominant phyla in the lid margin, whereas Proteobacteria, Bacteroidota, and Actinobacteria were found in the lid margin and eyelid skin. Differential expression revealed increased *Corynebacterium* and *Pseudomonas* in the lid margin, whereas *Staphylococcus* was predominant in the eyelid skin of patients with SJS (Fig. 6).

## Discussion

Although we found similar phyla in the two groups, at the genera level, there was an increase in the relative abundance of opportunistic pathogens *Haemophilus* (phylum Proteobacteria), *Azotobacter*, and *Corynebacterium* (phylum Actinobacteria), and a decrease of *Acinetobacter* (phylum Proteobacteria) in SJS lid margins as compared to healthy non-keratinized lid margins (see Fig. 3C). The abundance of *Corynebacterium* in keratinized lid margins is similar to psoriasis, where the hyperkeratinized skin shows an increase in *Streptococcus*, *Staphylococcus*, and *Corynebacterium.*^15^^,^^16^ Altered microbiota was postulated as one of the risk factors for LMK; however, it is difficult to say if the difference in microbiota of keratinized lid margins is an effect or a cause of keratinization. All patients clinically had obstructive MGD with keratinization, but the microbiota was different from that of patients with non-SJS MGD, except for the increase in *Corynebacterium.*^3^^,^^4^ *Corynebacterium* is a Gram-positive aerobic bacillus ubiquitously found on the skin and in the respiratory and gastrointestinal tracts. The keratinized lid margin shares a similar phenotype and cytokeratin expression as of the adjacent eyelid skin; hence, it is likely that the increase of *Corynebacterium* is due to epithelium phenotype rather than a causative factor for keratinization.^17^ The keratinized lid margin microbiota differs from adjacent eyelid skin in patients with SJS, where *Corynebacterium* was more abundant in the former and *Staphylococcus* was more abundant in the latter. The other explanation is that an altered microenvironment could be conducive to the growth of *Corynebacterium*.

It is well-established that conjunctival and tear film microbiome is altered in patients with SJS, but changes in lid margin microbiota were not studied before.^6^^,^^18^ Inferior conjunctival scrapings (avoiding the lid margin area) from 41 eyes of 22 patients with SJS showed Gram-positive cocci (*Streptococcus spp*. and *Staphylococcus aureus*) in 35 eyes, Gram-positive bacilli (*Corynebacterium* spp.) in 12 eyes, and Gram-negative bacilli (*Enterobacter spp*., *Serratia nonliquefaciens*, *Escherichia coli*, *Morganella morganii*, *Proteus mirabilis*, and *Haemophilus* spp.) in 16 eyes on routine culture alone.^7^ Ocular surface keratinization was reported in four patients (4/22) in their series. The current study evaluated microbiota (based on NGS) in keratinized lid margins, wherein not *Staphylococcus* but *Streptococcus* were found predominant in patients with SJS (using NGS). *Acinetobacter spp*., *Staphylococcus spp*., *Pseudomonas spp*., and *Streptococcus spp*. have been shown abundant in the conjunctiva of individuals with SJS.^5^^–^^10^ Methicillin-resistant *Staphylococcus aureus* was isolated in four patients with SJS.^9^ As a majority of these patients use contact lenses, *Staphylococcus* spp. was the dominant bacteria in 80% of individuals. However, there were no contact lens users in the current study. Low alpha diversity in patients with SJS was postulated to indicate these pathogenic organisms’ involvement in ocular surface inflammation. Our study also showed low alpha diversity. Although *Lactobacillus spp.*, *Prevotella spp.*, and *Fusobacterium spp*. have been reported in the SJS ocular surface, we did not find any increased prevalence of these microorganisms in the keratinized lid margins. A systematic review of the skin microbiome in psoriasis (skin hyperkeratinization) reported increased relative abundance of Firmicutes and lower relative abundances of Actinobacteria.^15^^,^^16^ At the genera level, there was a trend for an increase in *Streptococcus*, *Staphylococcus*, and *Corynebacterium* and a decrease in *Propionibacterium* compared to healthy controls.^16^ We observed a decrease in *Propionibacterium* in keratinized lid margins and a higher relative abundance of *Corynebacterium*; an increase in Actinobacteria and Proteobacteria phyla was present, contrary to the increase of Firmicutes only in psoriasis. The current study compared the adjacent eyelid skin with the lid margin microbiome and found distinct differences. There was a decrease in *Staphylococcus* and an increase in *Corynebacterium* prevalence in keratinized lid margins compared to the eyelid skin. Although the cytokeratin expression is similar in adjacent eyelid skin and lid margin, the microbiota differs.

Lid margin swabs cultured using conventional microbiological culture techniques show similar bacteria in individuals with and without MGD.^2^ Using NGS, meibum from patients with severe MGD showed predominant *Staphylococcus aureus* and *coagulase-negative Staphylococcus*, *Corynebacterium*, and *Lysinibacillus* sp. *Corynebacterium macginleyi* was only detected in the meibum of the patients with severe MGD group with an isolation rate of up to 26.3.^3^ However, they sterilized the (group 1 = healthy lid margins and group 2 = SJS lid margins) lid margin with povidone-iodine and only collected the meibum for microbiome study. Another study on MGD meibum (no lid margin pre-sterilization) showed an abundance of *Pseudomonas*, *Cutibacterium*, *Campylobacter*, *Corynebacterium*, and *Rubrobacter* genera that were also seen in eyelid skin and conjunctival microbiome of patients with MGD (Table). Other than *Corynebacterium* and *Pseudomonas*, there was no similarity between LMK microbiome and MGD meibum microbiome (Table). Interestingly, the network analysis in the LMK group predicted a major hub of negative interactions of the opportunistic pathogenic genera *Corynebacterium* and *Sphingomonas* with other bacterial genera (see Fig. 4). This indicates that opportunistic pathogens would compete for resources with other bacterial genera due to the prevailed imbalance in the microbiome, which was otherwise not possible in healthy conditions. The top pathogens that were significantly more prevalent in MGD meibum were *Campylobacter coli*, *Campylobacter jejuni*, and *Enterococcus faecium.*^4^ LMK completely covers the orifices of meibomian glands and does not let any meibum out. Hence, the meibum pathogens of MGD are unlikely to be seen in the LMK margins. The current study did not analyze the meibum for obvious reasons of no gland expressibility. This reiterates the possibility that lid margin microbiome changes are secondary to the changed epithelium phenotype rather than involved in LMK pathogenesis.

Normal conjunctival fornix has a diverse microbiota.^19^^,^^20^ Healthy conjunctival microbiota has 12 genera: *Pseudomonas*, *Propionibacterium*, *Bradyrhizobium*, *Corynebacterium*, *Acinetobacter*, *Brevundimonas*, *Staphylococci*, *Aquabacterium*, *Sphingomonas*, *Streptococcus*, *Streptophyta*, and *Methylobacterium* found in all specimens.^20^ Conjunctival microbiota demonstrates differences in context to the location within the ocular surface; *Corynebacterium* was found more in the skin and lid margin compared to the ocular surface (*Acinetobacter* and *Aeribacillus*).^11^ Inferior forniceal conjunctival swabs from 20 patients with SJS demonstrated a high proportion of pathogenic bacteria compared to healthy controls. The identified phyla were Proteobacteria (34.80%), Firmicutes (23.80%), Bacteroidetes (13.10%), Tenericutes (11.9%), and Actinobacteria (9.8%).^6^ Abundance significance plot showed *Lactobacillus*, *Bacteroides*, *Pseudomonas*, *Staphylococcus*, *Streptococcus*, *Bacillus*, and *Acinetobacter* to be higher in patients with SJS than healthy individuals.

There is an interplay of multiple factors on the ocular surface in SJS, including inflammation, dryness, limbal stem cell deficiency, goblet cell loss, and surface keratinization. The microbiota is expected to be altered, but its contribution to the LMK has never been studied. In addition, topical medications can alter the microbiota, as these patients require multiple medications, however, none of the patients were using steroid drops in the current study when sampled. The current study did not include or compare it with conventional culture techniques as the sampling site was small. The sample amount obtained from the lid margin alone without touching the adjacent structure is small, which explains the four samples that failed the quality check. Another limitation of this study is that the samples were not compared to the SJS eyelids without LMK. This is because the majority of patients with SJS at tertiary care have LMK, and, hence, it was difficult to obtain patients with SJS without LMK at our referral eye care center. The study's strengths are molecular amplification of 16S rDNA for identifying bacterial phyla and genera, which is extremely sensitive and useful in understanding the overall bacterial diversity. Future studies can look to see if there are any differences in microbiota based on the SJS etiology, patients with SJS with and without LMK, and the impact of eye drops.

In conclusion, this study's findings showed that the lid margin microbiome is significantly altered in the keratinized lid margins of patients with SJS compared to healthy lid margins, SJS eyelid skin microbiome, and patients with MGD. These results challenge the hypothesis that obstructive MGD is the cause of LMK or that microbiome changes associated with MGD have a causative role in LMK. Recent studies on histopathological and cytokeratin analysis of the lid margin epithelium in SJS eyes with LMK have strengthened the role of dermalization or inflammation-driven squamous metaplasia as the possible cause.^1^^,^^17^

## Supplementary Material

Supplement 1

Supplement 2

Supplement 3

## References

[1] Singh S, Jakati S, Shanbhag SS, Elhusseiny AM, Djalilian AR, Basu S. Lid margin keratinization in Stevens-Johnson syndrome: review of pathophysiology and histopathology. *Ocul Surf*. 2021; 21: 299–305. 33823305 10.1016/j.jtos.2021.03.011

[2] Watters GA, Turnbull PR, Swift S, Petty A, Craig JP. Ocular surface microbiome in meibomian gland dysfunction. *Clin Exp Ophthalmol*. 2017; 45(2): 105–111. 27473509 10.1111/ceo.12810

[3] Jiang X, Deng A, Yang J, et al. Pathogens in the Meibomian gland and conjunctival sac: microbiome of normal subjects and patients with Meibomian gland dysfunction. *Infect Drug Resist*. 2018; 11: 1729–1740. 30349330 10.2147/IDR.S162135 PMC6188152

[4] Zhao F, Zhang D, Ge C, et al. Metagenomic profiling of ocular surface microbiome changes in meibomian gland dysfunction. *Invest Ophthalmol Vis Sci*. 2020; 61(8): 22. 10.1167/iovs.61.8.22 PMC7425691 32673387

[5] Venugopal R, Satpathy G, Sangwan S, et al. Conjunctival microbial flora in ocular Stevens-Johnson syndrome sequelae patients at a tertiary eye care center. *Cornea*. 2016; 35(8): 1117–1121. 27124779 10.1097/ICO.0000000000000857

[6] Kittipibul T, Puangsricharern V, Chatsuwan T. Comparison of the ocular microbiome between chronic Stevens-Johnson syndrome patients and healthy subjects. *Sci Rep*. 2020; 10(1): 4353. 32152391 10.1038/s41598-020-60794-w PMC7062716

[7] Frizon L, Araújo MC, Andrade L, et al. Evaluation of conjunctival bacterial flora in patients with Stevens-Johnson syndrome. *Clinics (Sao Paulo)*. 2014; 69(3): 168–172. 24626941 10.6061/clinics/2014(03)04 PMC3935124

[8] Kittipibul T, Puangsricharern V. The ocular microbiome in Stevens-Johnson syndrome. *Front Med (Lausanne)*. 2021; 8: 645053. 34026783 10.3389/fmed.2021.645053 PMC8138458

[9] Sotozono C, Inagaki K, Fujita A, et al. Methicillin-resistant Staphylococcus aureus and methicillin-resistant Staphylococcus epidermidis infections in the cornea. *Cornea*. 2002; 21(7 Suppl): S94–S101. 12484707 10.1097/01.ico.0000263127.84015.3f

[10] Okonkwo A, Rimmer V, Walkden A, et al. Next-generation sequencing of the ocular surface microbiome: in health, contact lens wear, diabetes, trachoma, and dry eye. *Eye Contact Lens*. 2020; 46(4): 254–261. 32443013 10.1097/ICL.0000000000000697

[11] Ozkan J, Willcox M, Wemheuer B, Wilcsek G, Coroneo M, Thomas T. Biogeography of the human ocular microbiota. *Ocul Surf*. 2019; 17(1): 111–118. 30445178 10.1016/j.jtos.2018.11.005

[12] Suzuki T, Sutani T, Nakai H, Shirahige K, Kinoshita S. The microbiome of the meibum and ocular surface in healthy subjects. *Invest Ophthalmol Vis Sci*. 2020; 61(2): 18. 10.1167/iovs.61.2.18 PMC7326502 32053729

[13] Sotozono C, Ang LP, Koizumi N, et al. New grading system for the evaluation of chronic ocular manifestations in patients with Stevens-Johnson syndrome. *Ophthalmology*. 2007; 114(7): 1294–1302. 17475335 10.1016/j.ophtha.2006.10.029

[14] McKnight D, Huerlimann R, Bower DS, Schwarzkopf L, Alford RA, Zenger KR. microDecon: a highly accurate read-subtraction tool for the post-sequencing removal of contamination in metabarcoding studies. *Environmental DNA*. 2019; 1: 14–25.

[15] Alekseyenko AV, Perez-Perez GI, De Souza A, et al. Community differentiation of the cutaneous microbiota in psoriasis. *Microbiome*. 2013; 1(1): 31. 24451201 10.1186/2049-2618-1-31 PMC4177411

[16] Yerushalmi M, Elalouf O, Anderson M, Chandran V. The skin microbiome in psoriatic disease: a systematic review and critical appraisal. *J Transl Autoimmun*. 2019; 2: 100009. 32743498 10.1016/j.jtauto.2019.100009 PMC7388378

[17] Koduri MA, Jaffet J, Shanbhag SS, Basu S, Singh V, Singh S. Cytokeratin profile and keratinocyte gene expression in keratinized lid margins of patients with chronic Stevens-Johnson syndrome. *Graefes Arch Clin Exp Ophthalmol*. 2022; 260(9): 3009–3018. 35460363 10.1007/s00417-022-05669-8

[18] Pal S, Vani G, Donthineni PR, Basu S, Arunasri K. Tear film microbiome in Sjogren's and non-Sjogren's aqueous deficiency dry eye. *Indian J Ophthalmol*. 2023; 71(4): 1566–1573. 37026303 10.4103/IJO.IJO_2821_22 PMC10276756

[19] Huang Y, Yang B, Li W. Defining the normal core microbiome of conjunctival microbial communities. *Clin Microbiol Infect*. 2016; 22(7): 643.e7–643.e12. 10.1016/j.cmi.2016.04.008 27102141

[20] Dong Q, Brulc JM, Iovieno A, et al. Diversity of bacteria at healthy human conjunctiva. *Invest Ophthalmol Vis Sci*. 2011; 52(8): 5408–5413. 21571682 10.1167/iovs.10-6939 PMC3176057

