# Supplementary figures and images for: Lid Margin Microbiome in Stevens-Johnson Syndrome Patients With Lid Margin Keratinization and Severe Dry Eye Disease
Source: Invest Ophthalmol Vis Sci. 2024 Jun 18;65(6):28. doi: 10.1167/iovs.65.6.28 (PMC11193065; doi:10.1167/iovs.65.6.28)

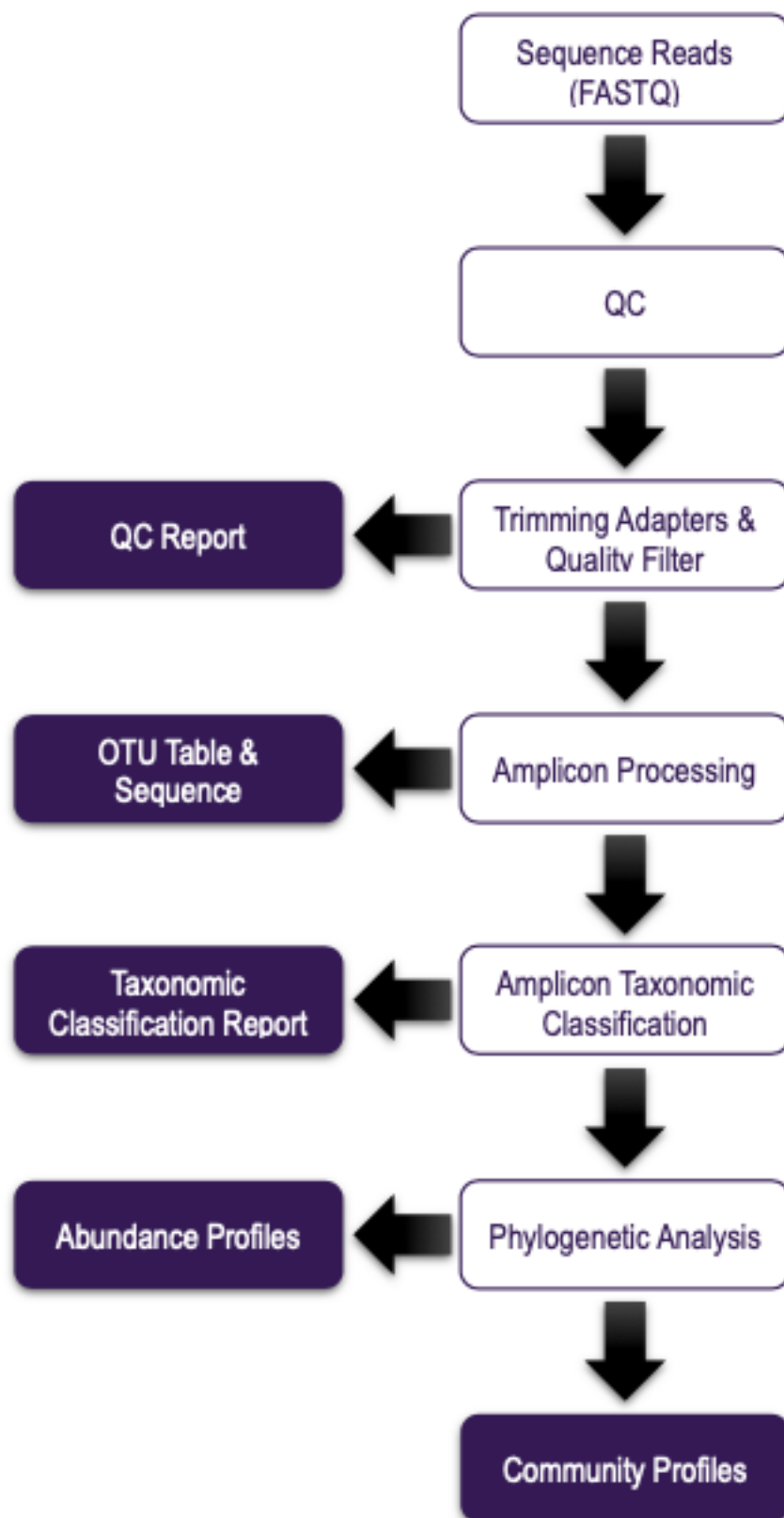

Supplementary File 1: Analysis workflow of the analyzed lid margin samples.

Supplement: Supplement 1 [file iovs-65-6-28_s001.pdf]

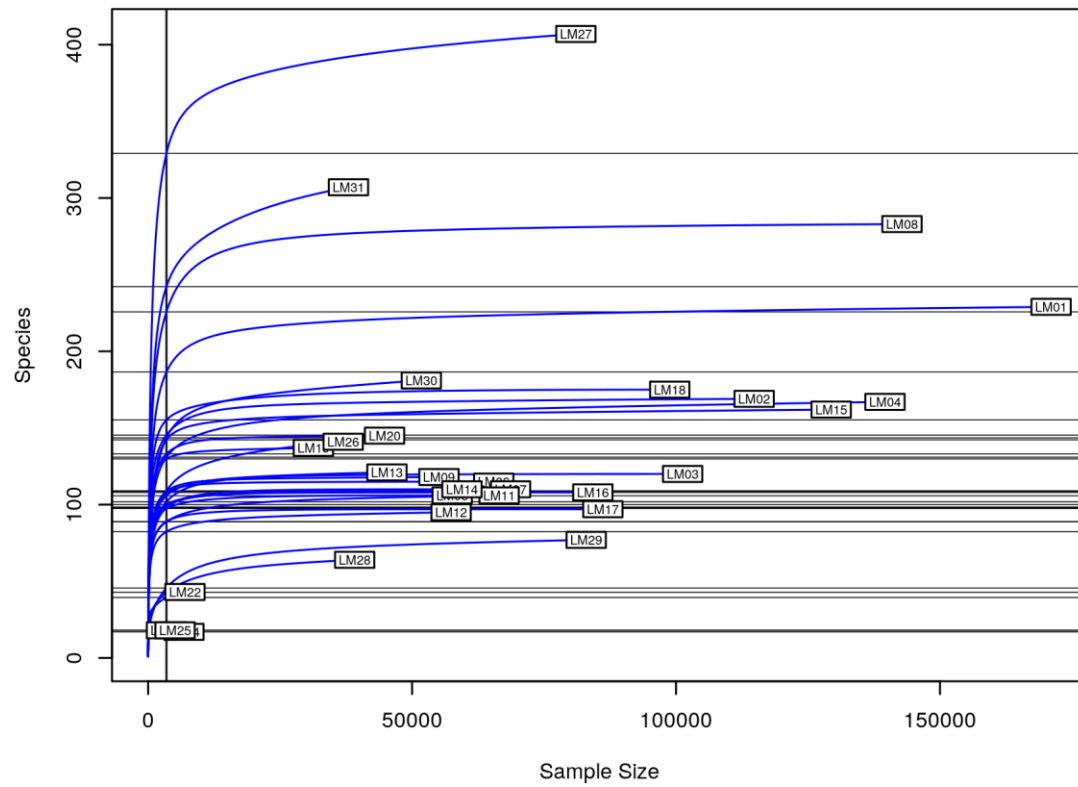

**Supplementary File 2:** Rarefaction curves of individual samples of both groups.

Supplement: Supplement 2 [file iovs-65-6-28_s002.pdf]
