# Supplementary material for: Lid Margin Microbiome in Stevens-Johnson Syndrome Patients With Lid Margin Keratinization and Severe Dry Eye Disease
Source: Invest Ophthalmol Vis Sci. 2024 Jun 18;65(6):28. doi: 10.1167/iovs.65.6.28 (PMC11193065; doi:10.1167/iovs.65.6.28)
Supplement: Supplement 3 [file iovs-65-6-28_s003.pdf]

Genera	log2FC	p_value
Bacillus	-7.4794	<0.001
Caulobacte	-5.58688	<0.002
Curvibacter	-3.26377	<0.003
Hydrotalea	-2.95782	<0.004
Lacunispha	-4.41426	<0.005
Sediminiba	-5.7243	<0.006
Staphyloco	-4.38334	<0.007
Afipia	3.648374	0.001
Bradyrhizol	-4.04927	0.001
Ga0074140	-2.15459	0.001
Novosphing	-4.61268	0.001
Reyranella	-3.27414	0.001
Microbacte	-2.52903	0.002
Pseudomor	-1.16874	0.002
Cricetibact	0.141522	0.004
Prevotellac	-3.10431	0.004
Ralstonia	-4.5716	0.004
Candidatus	-2.9729	0.012
Haemophil	4.784276	0.013
Corynebact	3.627119	0.023
Acinetobac	-1.14602	0.047
Aquamonas	0.847978	0.047
Azotobacte	3.446703	0.047
Oligotroph	2.989556	0.047
Pseudamin	2.768095	0.047
Stakelama	1.3405	0.047
Stenoxybac	0.7427	0.047

Supplementary File 3: Raw data of Log2 fold change in abundance of various genera between SJS (Group 2) versus healthy (Group 1) lid margins with P value.
